# A Multivariate Assessment of Age-Related Cognitive Impairment in *Octodon degus*

**DOI:** 10.3389/fnint.2021.719076

**Published:** 2021-08-30

**Authors:** Daniela S. Rivera, Carolina B. Lindsay, Carolina A. Oliva, Francisco Bozinovic, Nibaldo C. Inestrosa

**Affiliations:** ^1^GEMA Center for Genomics, Ecology and Environment, Facultad de Estudios Interdisciplinarios, Universidad Mayor, Santiago, Chile; ^2^Center of Aging and Regeneration UC (CARE-UC), Departamento de Biología Celular y Molecular, Facultad de Ciencias Biológicas, Pontificia Universidad Católica de Chile, Santiago, Chile; ^3^Center for Applied Ecology and Sustainability (CAPES), Departamento de Ecología, Facultad de Ciencias Biológicas, Pontificia Universidad Católica de Chile, Santiago, Chile; ^4^Centro de Excelencia en Biomedicina de Magallanes (CEBIMA), Universidad de Magallanes, Punta Arenas, Chile

**Keywords:** aging, cognitive performance, short-term memory, long-term memory, multivariate analysis, *Octodon degus*

## Abstract

Aging is a progressive functional decline characterized by a gradual deterioration in physiological function and behavior. The most important age-related change in cognitive function is decline in cognitive performance (i.e., the processing or transformation of information to make decisions that includes speed of processing, working memory, and learning). The purpose of this study is to outline the changes in age-related cognitive performance (i.e., short-term recognition memory and long-term learning and memory) in long-lived *Octodon degus*. The strong similarity between degus and humans in social, metabolic, biochemical, and cognitive aspects makes it a unique animal model for exploring the mechanisms underlying the behavioral and cognitive deficits related to natural aging. In this study, we examined young adult female degus (12- and 24-months-old) and aged female degus (38-, 56-, and 75-months-old) that were exposed to a battery of cognitive-behavioral tests. Multivariate analyses of data from the Social Interaction test or Novel Object/Local Recognition (to measure short-term recognition memory), and the Barnes maze test (to measure long-term learning and memory) revealed a consistent pattern. Young animals formed a separate group of aged degus for both short- and long-term memories. The association between the first component of the principal component analysis (PCA) from short-term memory with the first component of the PCA from long-term memory showed a significant negative correlation. This suggests age-dependent differences in both memories, with the aged degus having higher values of long-term memory ability but poor short-term recognition memory, whereas in the young degus an opposite pattern was found. Approximately 5% of the young and 80% of the aged degus showed an impaired short-term recognition memory; whereas for long-term memory about 32% of the young degus and 57% of the aged degus showed decreased performance on the Barnes maze test. Throughout this study, we outlined age-dependent cognitive performance decline during natural aging in degus. Moreover, we also demonstrated that the use of a multivariate approach let us explore and visualize complex behavioral variables, and identified specific behavioral patterns that allowed us to make powerful conclusions that will facilitate further the study on the biology of aging. In addition, this study could help predict the onset of the aging process based on behavioral performance.

## Introduction

From a lifespan perspective, aging is the progressive decline in physiological homeostasis and functional integrity, and is associated with a gradual reduction in the capacity of the brain to transmit signals and self-repair (Amarya et al., 2018; Singh et al., 2019). Given that, the brain can use different mechanisms to adapt itself considering the wear and tear that comes with the natural life history of every species (Erickson and Barnes, 2003; Besdine and Wu, 2008; Singh et al., 2019). Therefore, studying the effect of natural aging on cognitive and behavioral animal capacities is one of the major areas of interest in the field of neuroscience.

During natural aging, cognitive capacities such as the speed of processing, working memory, and long-term memory tended to decline more slowly and to a slighter degree than physical abilities (Erickson and Barnes, 2003; Caserta et al., 2009; Park and Reuter-Lorenz, 2009; Amarya et al., 2018). For example, naturally old mammals (e.g., aged rodents, primates, and humans) showed spatial memory impairments compared with their younger counterparts (Barnes, 1979; Uttl and Graf, 1993; Rapp et al., 1997; Bach et al., 1999). Furthermore, changes in memory with age do not occur linearly; instead, it can be variable between individuals, and not all kinds of memory are equally affected (Markowska et al., 1989; Rapp and Amaral, 1991, 1992; Erickson and Barnes, 2003; Rodefer and Baxter, 2007).

Evidence from animal models and humans showed that in addition to advanced age, multiple factors might contribute to cognitive performance declines such as high blood pressure, diabetes, poor smelling ability, higher homocysteine level, depression, coronary artery disease, stroke, and any type of systemic chronic illness (Selhub et al., 2000; Kruman et al., 2002; Lipnicki et al., 2013; Wiesmann et al., 2017; d'Avila et al., 2018; de Montgolfier et al., 2019). Environmental or occupational conditions and lifestyle factors could also have a significant impact on cognitive abilities (Hartman, 1987; Dupont-Frechette and Marraccini, 2014; Bhatt et al., 2015; Huang et al., 2016; Woods et al., 2016; Cacciottolo et al., 2017; Choi et al., 2017; Wahl et al., 2017). Moreover, genetic factors (e.g., apolipoprotein E ε4 allele) and hormonal level (e.g., cortisol, estrogen, thyroid, and pituitary hormones) have been associated with poorer cognitive performance, and this condition could be exacerbated by additional chronic disease (de Kloet et al., 2002; Small et al., 2004; Frick, 2009; Liu et al., 2013; Zhu et al., 2018; Christensen et al., 2020).

Given that memory loss is one of the key features of cognitive impairment in physiological aging, age-related cognitive decline directly affects several psychological domains crucial for daily functioning, such as attention and memory (Masdeu et al., 2012; Tarragon et al., 2013, 2014). Besides, the functional loss occurring with aging may also indicate other more severe pathological conditions arising, such as the development and progression of Alzheimer's disease (AD) and other forms of dementia (Tarragon et al., 2014; Weber et al., 2015). However, few but consistent differences in behavioral and physiological outcomes may distinguish these impairments (Tarragon et al., 2014). In this context, behavioral tests are performed to probe diverse aspects of learning and memory; they involve several brain regions required to orientate and navigate using different spatial information cues (Grech et al., 2018).

Appropriate animal models of aging are necessary to understand both the physiological and pathological mechanisms of age-related cognitive decline (Gallagher and Rapp, 1997; Holmes, 2004; Mitchell et al., 2015). The behavioral assessment of cognitive function in long-lived animal models provides a basis for understanding biological factors that contribute to cognitive impairments associated with natural aging (Levin and Buccafusco, 2006; Edrey et al., 2011; Yeoman et al., 2012; Stenvinkel and Shiels, 2019). The social rodent species *Octodon degus* (hereafter called degus) has become an increasingly popular animal model for exploring the mechanisms underlying behavioral and cognitive deficits resulting from aging disorders (i.e., age-related cognitive decline) (Tarragon et al., 2013). In this context, degus are characterized by their cognitive abilities, such as social and spatial recognition, which declines with aging similar to that observed in humans (Uekita and Okanoya, 2011; Tarragon et al., 2013). Degus live for an average of 7–8 years in captivity, making it an extraordinarily useful rodent model for longitudinal studies (Lee, 2004). For example, Rivera et al. (2018) demonstrated that long-term sugar consumption (from pups to adulthood) affected the normal aging process in degus, resulting in reduced synaptic plasticity and cognitive impairment upon reaching adulthood. Moreover, in another longitudinal study, the same group demonstrated that long-term chronic social isolation affected cognitive performance (Rivera et al., 2020) and social behavior (Rivera et al., 2021) in adult female and male degus. They showed that these effects have molecular correlates in brain-related regions involved in such activities, demonstrating that several aspects of neuronal and system physiology of degus are useful as an animal model.

Degus has also gained prominence as a valued model for many different diseases, such as those related to lipid metabolism and atherosclerosis (Homan et al., 2010), diabetes mellitus (Edwards, 2009), cataracts and retinal degeneration (Datiles, 1989; Brown and Donnelly, 2001; Du et al., 2015), cancer (Lester et al., 2005; Ardiles et al., 2013; Svara et al., 2020), and neurodegenerative disorders such as AD (Inestrosa et al., 2005; van Groen et al., 2011; Hurley et al., 2018). Aging degus spontaneously develop some neuropathological hallmarks of AD, such as Aβ accumulation, *tau* hyperphosphorylation, and cognitive impairments when reaching the age of 3–4 years (Inestrosa et al., 2005; Ardiles et al., 2012; Braidy et al., 2012; Deacon et al., 2015; Hurley et al., 2018). Indeed, have been found high similarities between human and degus ApoE, Amyloid β, and *tau* proteins (Salazar et al., 2016; Steffen et al., 2016; Hurley et al., 2018), reasons why degus is considered a natural model of AD-like pathology (Inestrosa et al., 2005; Cisternas et al., 2018).

The cognitive impairment observed during natural aging, in particular memory dysfunction, and other cognitive domains such as social behavior, personality change, orientation, or problem-solving, are also the main symptoms of several types of dementia and late-onset AD pathology. A previous study on degus showed a reduction in spatial memory and object recognition in aged animals (Ardiles et al., 2012). Related to this, the degus 56-months of age showed impaired cognitive performance in spatial and recognition memory tasks, together with a reduction in synaptic function compared with the young degus (12-months-old) (Rivera et al., 2016). A well-designed experiment on degus should be able to discriminate the different memory deficits classically associated with AD (Tarragon et al., 2013; Cisternas et al., 2018).

To gain a better understanding of the effects of natural aging on the cognitive capacity of degus, this study aimed to record behavioral patterns in specific behavioral tasks, assessing short-term performance in social novelty and recognition memory (Social Interaction and the Novel Local/Object Recognition tests), and learning and memory process (Barnes maze) across female degus of different ages. Using the information on cognitive performance of published and unpublished data obtained in the last 6 years from the laboratory, we performed a multivariate approach (principal component analyses of the data) to assess age-related cognitive impairment in this animal model and provide a more practical method to understand the functional importance of several parameters used in conventional behavioral tests.

## Materials and Methods

### Animals

We used data of behavioral tasks commonly used to assess cognitive abilities from a cohort of 39 adult female degus of different ages that were used as a control group across 6 years of experiments. The aging range varies from 12- to 75-months-old. The degus were grouped in five age categories: 12- (*n* = 6), 24- (*n* = 13), 38- (*n* = 6), 56- (*n* = 5), and 75-months-old (*n* = 9). We only included studies that reported equal maintenance conditions and the same protocols of behavioral tasks. In this context, all the animals were obtained from the colony at the Faculty of Biological Sciences, Pontificia Universidad Católica de Chile, kept in pairs, and housed in clear acrylic terrariums (length × height × depth: 50 × 35 × 23 cm) with hardwood chip bedding. Each cage contained one nest box made of clear acrylic (22 × 12 × 15 cm). All the animals were kept in a ventilated room and exposed to a 12L:12D and ambient temperature (yearly minimum = 13.4 ± 0.2°C; yearly maximum = 24.9 ± 0.2°C). The degus were fed a standard rabbit commercial pellet diet (Champion, Santiago, Chile) and *ad libitum* water. All the animal protocols followed the guidelines of the National Institutes of Health (NIH, Baltimore, MD, United States). All the procedures were approved by the Bioethical and Biosafety Committee of the Faculty of Biological Sciences of the Pontificia Universidad Católica de Chile (CBB-121-2013).

### Behavioral Tests

The behavioral information of this study included data obtained from the Open Field, the Social Interaction, Novel Local and Novel Object Recognition (NLR/NOR), and Barnes maze tests. From the published data, we used 6 12-months-old, 13 24-months-old, and 5 56-months-old female degus (Rivera et al., 2016, 2020, 2021). From the unpublished data, we used six of 12-months-old, six 38-months-old, five 56-months-old, and nine 75-months-old female degus. Exclusion criteria are applied when the animal shows an evident motor dysfunction or defective eyes (e.g., cataracts). We did not find any of these conditions in our animal groups. Moreover, in the understanding that aged animals could eventually die, none of our animals died during the study time.

We used data from the Open Field test to discard any locomotor differences across age groups. This test consists of animal observation within a white Plexiglas box (length × height × depth: 100× 100× 100 cm). The frequency of central crossings (with a four-paw criterion), the percentage of time in the corners and in the middle arena, the total distance traveled, and speed were assessed (Rivera et al., 2021). The Social Interaction test was performed to evaluate the social memory and preference for social novelty (Kaidanovich-Beilin et al., 2011). The NLR/NOR is a double test performed to evaluate cognition, particularly working memory and attention, but it can also be used to test the preference for novelty in rodents (Popovic et al., 2009). Both behavioral tests measure the ability to recognize an unfamiliar vs. a familiar partner, or a familiar object vs. a novel object, respectively (Dere et al., 2007; Kaidanovich-Beilin et al., 2011). For the Social Interaction test, the female degus were exposed to a 20-min habituation session to the area and then tested in two consecutive 20-min sessions with a 1-h inter-session interval resting in the animal home cage (Rivera et al., 2021). For the NLR/NOR, the animals were exposed to a 10-min familiarization session and then tested in two consecutive 5-min sessions, with a 1-h inter-session interval resting in the animal home cage (Rivera et al., 2020). Both tests last < 1 day, where the 1-h interval between sessions is the time for memory consolidation. Therefore, the information of both tests evaluates the short-term recognition memory (hereafter called short-term memory).

On the other hand, we used the Barnes maze data to evaluate long-term learning and memory processes (hereafter called long-term memory). The Barnes maze is a dry-land-based behavioral test by which animals learn the relationship between distal cues in the surrounding environment and a fixed escape location (Barnes, 1979; Pitts, 2018). Following a sufficient acquisition training period, the degus were submitted to a probe trial to evaluate spatial navigation, learning, and memory. Briefly, the procedure was divided into three phases: habituation, training, and test. The training period consisted of four consecutive 4-min trials, separated by a 5-min resting phase in the animal home cage for 7 consecutive days. At the end of the training period, the animals were left resting for 7 days, and then they were exposed to a memory-retrieval session (four consecutive 4-min trials, separated by a 5-min resting phase in the animal home), see Rivera et al. (2020). Because of the length of memory retention required, the Barnes maze was used to measure long-term memory.

### Multivariate Analysis

Across the study of animal behavior, animals are often made to undergo a battery of tests to gain comprehensive results for different behavioral patterns. In most cases, the information from these behavioral tests is analyzed by a univariate approach (e.g., ANOVA, Student's *t*-test); however, to explore and visualize behavioral variables of different tests and make the assignment to underlying behavioral patterns easier, the multivariate approach is suggested (Feyissa et al., 2017).

To reduce the high number of variables reported by each behavioral test to a lower number of representative factors, we performed principal component analysis (PCA) with orthogonal rotation (Feyissa et al., 2017; Matzel and Sauce, 2017). The orthogonal rotation provides independence of the factors, and the behavioral variables with high factor specific loadings, from each other (Feyissa et al., 2017). In addition, the PCA used the correlation matrix because the variables were on different scales, and this approach standardized the data (Jolliffe and Cadima, 2016).

The first component is the linear combination of all studied variables that result in the maximum variance (among all linear combinations), so it explains most of the variance in the data as possible (expressed in terms of the first eigenvalue). The second component is the linear combination of all studied variables that accounts for as much of the remaining variation. The third component follows the same logic, and so forth. The number of possible principal components will vary depending on the number of the studied variables. The loading of each measure on a principal component represents the correlation between the latent characteristic and the original measure and, thus, indicates the importance of a measure for a principal component. Measures with high loadings on the same principal component of the same sign are positively correlated, and loadings of the opposite sign are negatively correlated. An eigenvalue > 1 was set as the criterion for selecting components (Feyissa et al., 2017).

By principal component analysis, first, we discarded differences in the locomotor activity and the willingness of the exploratory behavior between the aging groups. Then, we used the time that animals spent in the central zone, the time spent in the corners, the number of central crossings, the total distance traveled, and the speed during the Open Field test. For short-term memory analyses, we used the Recognition Index information (RI) of both the Social Interaction and the NLR/NOR tests. For long-term memory, the PCA included information of latency to the first visit of the escape hole, the reference memory errors (i.e., every first visit of a non-escape hole in each trial), and working memory errors (i.e., repeated visits to the same non-escape hole in the same trial) to find the escape hole (Rivera et al., 2020).

### Statistical Analysis

To assess the statistical significance of each age group across principal component analysis for both short-term and long-term memories, we performed a one-way permutational multivariate ANOVA (PERMANOVA) (9,999 permutations), which permuted the distance matrix (Euclidian method) (Anderson and Ter Braak, 2003). For the training period of the Barnes maze, we performed two-way PERMANOVA, with age as the first factor and the time of training as the second factor. In addition, we assessed the association between the first axis of PCA from the short-term memory analysis with the first axis of PCA from long-term memory using Spearman' rank correlation coefficient. This analysis may help to evaluate the position of the data points in the coordinate space of the principal components revealing the strength of the association pattern between short and long-term memories in the young and aged degus. Finally, we performed one-way ANOVA to analyze the effect of aging across each variable of the behavioral test. Multivariate analyses were performed using the program CANOCO (Ter Braak and Smilauer, 1998), whereas univariate analyses were performed using the Statistica software package (StatSoft, Tulsa, OK, United States). Differences were considered statistically significant at *p* < 0.05.

## Results

### General Cognitive Performance in a Long-Lived Animal Model

The main results of the principal component analysis for the five measures of the Open Field test produced two components with eigenvalues greater than 1. These two components explain 78% of the variance in the correlation matrix. The rotated factor patterns are presented in Supplementary Table 1 (Supplementary Material, SM). The first component explained 58.3% of the total variance and was mainly loaded by the total distance traveled and speed (both = 0.8), time spent in the central zone (0.77), time spent in the corners (−0.73), and the number of central crossings (0.52); whereas the second component explained 20.2% of the variance and was loaded by the time spent in the corners (0.55), time spent in the central zone (−0.5), total distance traveled, and speed (both = 0.47). The one-way PERMANOVA test for the PCA of the Open Field test showed no significant differences between the aging groups (*p* > 0.05), suggesting that despite aging all the animals exhibited normal motor activity (Supplementary Figure 1; SM).

**Figure 1:**
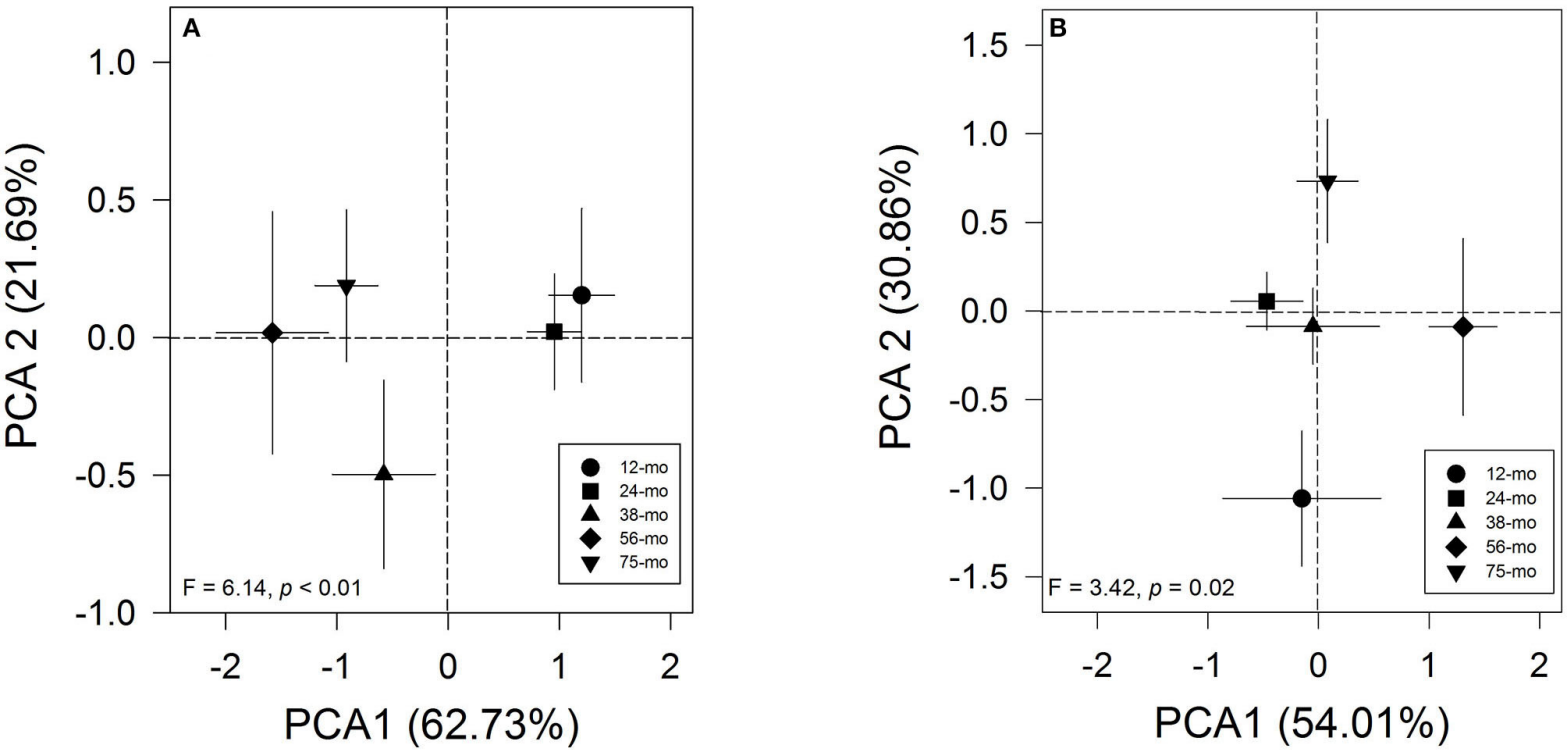
Principal component analysis graph of **(A)** short-term memory and **(B)** long-term memory processes across age groups. Each symbol represents the age of animals (circle: 12-months-old (*n* = 6 per group); square: 24-months-old (*n* = 13 per group); triangle: 38-months-old (*n* = 6 per group); diamond: 56-months-old (*n* = 5 per group); inverted triangle: 75-months-old (*n* = 9 per group). For short-term memory analysis, we used Recognition Index information (RI) from the Social Interaction test and the Novel Local Recognition/Novel Object Recognition test. For long-term memory analysis, the principal component analysis (PCA) included information of latency to the first visit of the escape hole and the reference and working memory errors to find the escape hole during the trials of the Barnes maze test. The *F* and *p* values of the one-way PERMANOVA test are plotted.

The results of the principal component analysis of short-term memory produced only one component with eigenvalues > 1. This component explains 62.7% of the variance in the correlation matrix. The rotated factor patterns are presented in Supplementary Table 2 (SM). This component was positively highly loaded by the RI of the NOR test (0.84), RI of the social memory in the Social interaction test (0.79), and RI of the NLR test (0.75). In this case, the short-term memory *via* PCA showed a clear pattern between age groups (Figure 1A). Likewise, the one-way PERMANOVA test confirmed that the young 12- and 24-months-old degus formed a separate group from the 38-, 56-, and 75-months-old animals (*F* = 6.14, *p*<0.01; Table 1). Importantly, these results reflect a strong pattern on the effect of the aging process on the short-term memory of adult female degus. The two-way PERMANOVA test for the long-term memory during the training period of the Barnes maze showed a significant effect of the age group [*F*_(4, 272)_ = 163.64, *p*<0.01] but was not altered by the time of training (*p* = 0.06), and there was a significant interaction between both factors [*F*_(24, 272)_ = 0.56, *p* < 0.01]. The main results of the PCA for long-term memory yielded two components, cumulatively explaining 92.9% of the total variation. For the test phase of the Barnes maze test, the rotated factor patterns are presented in Supplementary Table 3 (SM). The first component explained 52.3% of the total variance and was positively loaded by the working memory errors (0.92), the reference memory errors (0.84), and the first visit of the escape hole (0.14); whereas, the second component (40.7% of variance) was loaded by the first visit of the escape hole (0.96), the reference memory errors (−0.46), and the working memory errors (0.27). In this case, the long-term memory *via* PCA showed a clear pattern between age groups (Figure 1B). Furthermore, the one-way PERMANOVA test revealed a significant pattern across age groups (*F* = 3.42, *p* = 0.02; Figure 1B) where only the 12-months-old degus were statistically different from those 56- and 75-months-old (Table 1). These results suggest that natural aging affects the long-term memory of the female degus.

**Table 1:** One-way permutational multivariate ANOVA (PERMANOVA) test of age-related cognitive performance for short-term memory (values below diagonal line) and long-term memory (values above diagonal line).

**Age**	**12 months**	**24 months**	**38 months**	**56 months**	**75 months**
12 months	–	0.0552	0.1564	**0.0135**	**0.0076**
24 months	0.8318	–	0.8958	0.0852	0.0791
38 months	**0.0038**	**0.0011**	–	0.1579	0.1650
56 months	**0.0027**	**0.0005**	0.1514	–	0.7075
75 months	**0.0004**	**0.0002**	0.3891	0.3992	–

*The values in bold indicate statistical significance at p < 0.05 (9,999 permutations)*.

**Figure 2:**
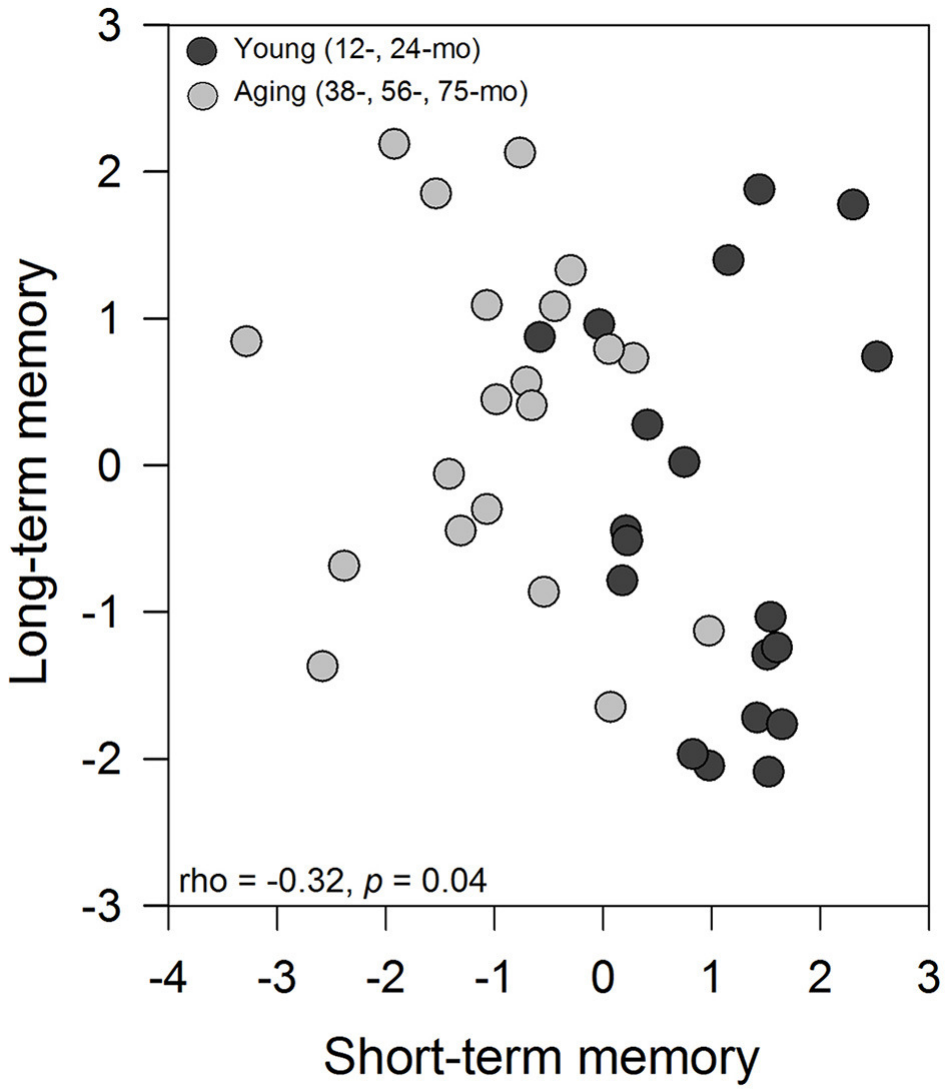
Association between the first axis of PCA from the short-term memory analysis with the first axis from long-term memory using Spearman' rank correlation coefficient. Each data point represents a degus, and symbols represent the age group of animals (black circle: young degus (12- and 24-months-old); gray circle: aged degus (38-, 56-, and 75-months-old).

The Spearman' rank correlation coefficient between the first axis of principal component analysis from short-term memory with the first axis of principal component analysis from long-term memory indicated a negative and significant relationship between variables (rho = −0.32, *p* = 0.047; Figure 2). A clear pattern emerges from this analysis, with the young animals showing significantly higher values for short-term memory and lower values for long-term memory. In general, a clear behavioral pattern emerges from this analysis, with the young animals showing significantly higher values for short-term memory and lower values for long-term memory. In contrast, the aged degus exhibited lower values for short-term memory but higher values for long-term memory.

### Age-Related Cognitive Performance in a Long-Lived Animal Model

**Figure 3:**
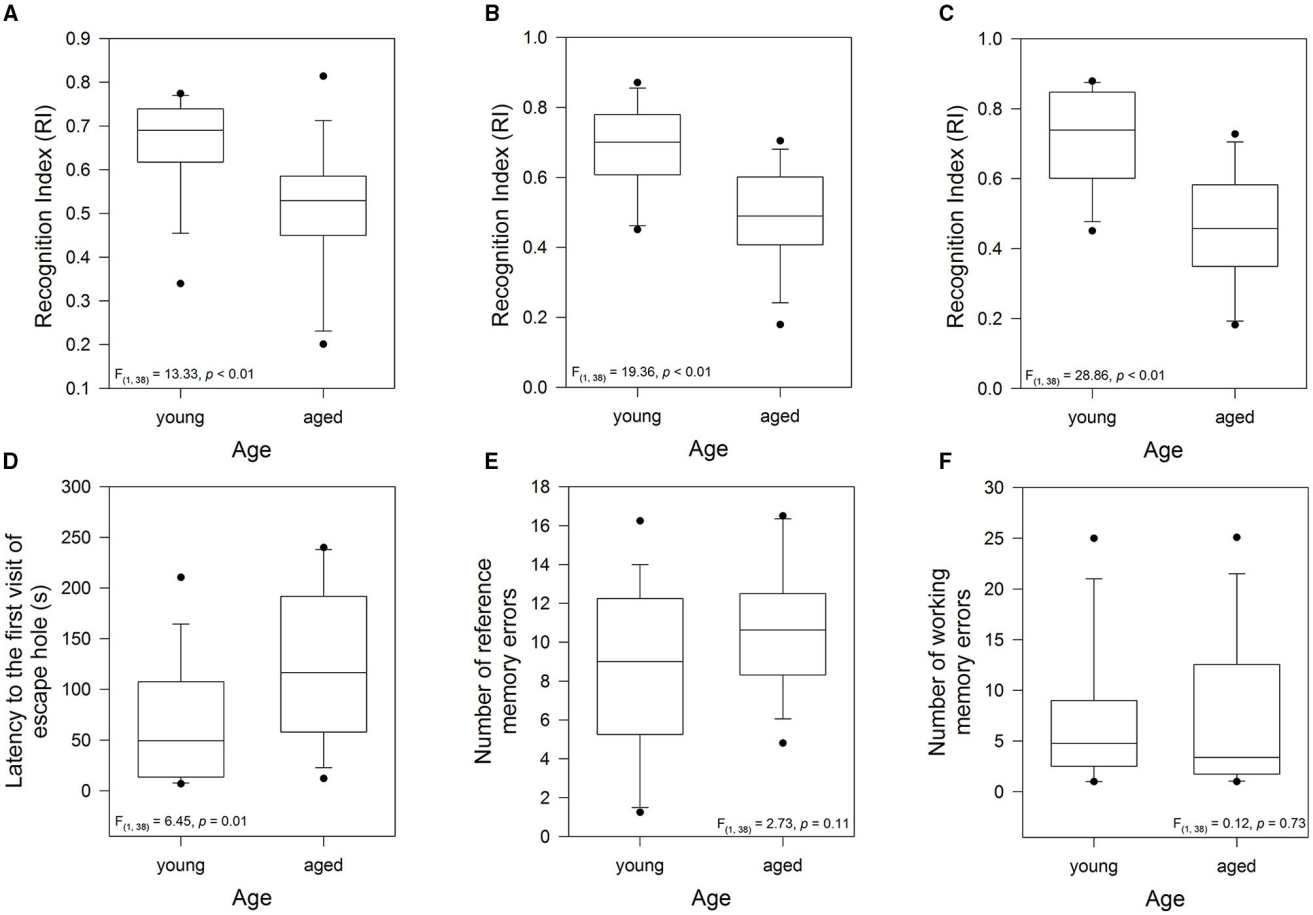
Evaluation of the cognitive performance of young group (12-, and 24-months-old, *n* = 19) and aged group (38-, 56-, and 75-months-old, *n* = 20): short-term memory measured by **(A)** the Recognition Index (RI) of the Social Interaction test, **(B)** the RI of the novel local recognition test, and **(C)** the RI of the novel object recognition test. Long-term memory measured during the test phase of the Barnes maze: **(D)** latency to the first visit of the escape hole, **(E)** reference memory errors to find the escape hole (every first visit of a non-escape hole in each trial), and **(F)** working memory errors (repeated visits to the same non-escape hole in the same trial). The data were analyzed statistically by one-way ANOVA followed by Tukey's *post hoc* test.

The above age pattern allowed us to classify the animals as young (12- to 24-months-old) and aged (38- to 75-months-old). In this context, to evaluate the effect of aging on short-term memory of the female degus, we evaluated the exploratory motivation of the degus to interact with an unfamiliar (novel) vs. familiar degus in the Social Interaction test, and the time spent interacting with a novel object in the NLR/NOR test. The one-way ANOVA on the Social Interaction test showed that the aged degus had lower values of RI compared with the younger group [*F*_(1, 37)_ = 13.33, *p* < 0.01; Figure 3A]. This result suggests that short-term social memory was impaired in the aged degus. Similarly, the RI for the NLR/NOR tests revealed that the values for the aged animals were significantly lower than those for the younger ones [*F*_(1, 37)_ = 19.36, *p* < 0.01 and *F*_(1, 37)_ = 28.86, *p* < 0.01; respectively; Figures 3B,C]. Taken together, these results showed that the young animals have higher values of RI (greater exploration of the unfamiliar partner, novel location, or object, indicates remembering abilities) compared with the aged animals. In addition, short-term memory was impaired in the old-aged degus.

To examine the effect of aging on long-term memory, we analyzed the performance during the test phase of the Barnes maze test. In this context, the time taken to find the escape hole, a measurement of long-term memory, was significantly higher in the aged animals [one-way ANOVA, *F*_(1, 37)_ = 6.45, *p* = 0.015; Figure 3D]. No statistically significant differences were recorded in both the reference memory and the working memory errors (*p* = 0.11 and *p* = 0.73; respectively; Figures 3E,F). These results indicated that during the retention phase, the latency to the first visit to the escape hole, one of the most widely used measures of learning in the Barnes maze, was the most sensitive for detecting differences between the young and aged degus. Thus, long-term memory was impaired in the aged group.

## Discussion

Aging is a progressive functional decline characterized by gradual deterioration in physiological function and performance behavior (Shoji and Mizoguchi, 2010; Lopez-Otin et al., 2013), which is responsible for the increased risk of disease and death (Galluzzi et al., 2008). Consequently, it is of particular interest to study the natural aging process to determine at what point aging begins and becomes irreversible. Understanding the potential mechanisms that underlie these age-related impairments would allow us to target, slowdown, or even reverse them.

This study showed natural age-related impairments in the long-lived *Octodon degus* across a series of behavioral tasks that evaluated short-term and long-term memories. We used both published and non-published data from trials of Session 2 of the Social Interaction test and NLR/NOR test to assess short-term recognition memory in young and aged degus. Both tests are novelty-preference paradigms aimed to assess recognition memory, and both require innate motivation to explore novel stimuli (Bevins and Besheer, 2006; Dere et al., 2007; Kaidanovich-Beilin et al., 2011). Both tests were evaluated using the Recognition Index and showed that the aged animals exhibited lower values for this index than the younger ones.

For the long-term memory, we used published and unpublished data from trials of the Barnes maze test. This test assesses spatial learning and memory in rodents and has a strong hippocampal-dependent spatial component (Barnes, 1979; Kennard and Woodruff-Pak, 2011; Negron-Oyarzo et al., 2015; Pitts, 2018). The Barnes maze test assumes that the animal placed into the aversive environment should learn and remember the location of an escape box located below the surface of the platform (Pitts, 2018; Gawel et al., 2019). The usefulness of this task includes assessing the outcome in neurodegenerative disease (e.g., AD, Parkinson's disease) and postoperative dysfunction of cognition (e.g., traumatic brain injury), as well as drug regimens that might improve or deteriorate the long-term spatial learning and memory process (Gawel et al., 2019).

First, we performed a multivariate analysis to identify patterns across several variables of the different behavioral tests. In general, the study on age-related cognitive dysfunction can be difficult because of changes in sensory or locomotor function (Markowska, 1999). Therefore, we first discarded any locomotor dysfunction across the five age groups as evaluated by the Open Field variables. The PCA for short-term memory revealed a clear pattern in the cognitive ability distribution across the five age categories, with the young group (12-and 24-months-old) displaying better cognitive abilities than the aged group (38-, 56-, and 75-months-old). In this context, recognition memory comprises both familiarity detection and recollection of information (Aggleton and Brown, 2006), two functions that have substantial postnatal development and reorganization in rats, monkeys, and humans (Reger et al., 2009).

The results are in agreement with those of previous behavioral studies using animal models which showed that aging impaired the ability to discriminate novel stimuli from those previously introduced (de Lima et al., 2005; Pitsikas et al., 2005; Murai et al., 2007; Pieta Dias et al., 2007; Insel et al., 2008; Burke et al., 2010). Similarly, a decrease in social recognition memory in aged rodents has also been shown (Prediger et al., 2005; Markham and Juraska, 2007). In degus, Ardiles et al. (2012) reported that aged degus did not demonstrate a preference between the novel and familiar objects (Ardiles et al., 2012). Similarly, the previous results in young and aged female degus indicated that aged animals are less explorative than the younger ones who always are more willing to spend time exploring when the stimulus is novel (Rivera et al., 2016). Moreover, aged female degus showed impaired social memory compared with young animals (D.S. Rivera, unpublished data).

The effect of aging on long-term learning and memory has been intensively studied in rats and mice by the Barnes maze test. The results of PCA showed that only the 12-months-old degus group was different from the 56- and 75-months-old groups. Also, senescent Long–Evans rats (28- to 34-months-old) showed impairment in all the Barnes maze-dependent measurements (Barnes, 1979) compared with younger animals (10- to 16-months-old). Similarly, aged rodents, such as Sprague–Dawley and Dark Agouti, showed cognitive impairment including latency to reach the escape hole and the number of errors made (Barnes, 1979; Barnes et al., 1980; McLay et al., 1999; Barrett et al., 2009; Barreto et al., 2010). Cognitive dysfunction has been also reported in aged strains of mice (e.g., C57BL/6 mice) that made more errors than the young ones (Bach et al., 1999). In degus, behavioral experiments showed that aged degus had poor performance compared with the younger in the T-maze test (Ardiles et al., 2012). Furthermore, Rivera et al. (2016) reported deficits in the spatial memory of aged female degus measured by the latency to the first visit of the escape hole (aged degus required approximately five-fold longer time to locate the escape hole compared with younger animals) (Rivera et al., 2016). Similar to the results of this study, no differences were found in the number of reference and memory errors. The reason for the latter may be that most rodents are more likely to explore other holes instead of entering the escape hole, even when they found the location of the escape hole (Grootendorst et al., 2001). Therefore, between young and old animals, the consensus is that the former performs better in long-term memory tasks than the latter.

Despite this not following the trend, there is evidence showing that some old animals can remain cognitively young, suggesting consideration for the inter-individual variability associated with cognitive changes related to age. Studies comparing young and aged mice performance with an object recognition task showed that both behaved similarly, suggesting that hippocampal-independent memories would be unaffected by the aging process (Wimmer et al., 2012). Vogel et al. examined the development of age-related cognitive impairments in C57BL/6 mice. In this study, the performance of aged mice in the hippocampal-dependent Morris water maze task was comparable to that of younger animals (Vogel et al., 2002). Similar studies showed that the performance of aged mice in the water maze was similar to that of young ones, but that the former used a different strategy for searching (von Bohlen Und Halbach et al., 2006). These data suggest that some neuronal mechanisms, brain region or circuit dependent, could be selectively affected in the aging process. Humans are not an exception. Some elderly adults retain excellent cognitive function even in their 70s and 80s; their performance becomes similar to or better than that of younger adults, whereas other adults showed signs of decline starting as early as 60 (Glisky, 2007). In a very recent study, a group of aged adults displayed learning and recalling abilities similar to 25 years old individuals; they have been called “superagers”. Better encoding mechanisms that lead to successful memory retrieval appear to differentiate them from average older adults (Katsumi et al., 2021). On the other hand, an increase in brain activation in aged and younger adults while performing identical memory tasks has been reported (Cabeza et al., 2002; Grady, 2002). In a visual short-term memory task, positron emission tomography (PET) measurements showed that older participants performed equally with younger individuals. In this study, the older participants showed a weaker communication of brain areas when compared with those used by the younger ones; however, the former recruited different brain areas to compensate (McIntosh et al., 1999; Cabeza et al., 2002). These effects are attributed to some class of compensatory activity, supporting some reorganization of the aged brain that leads to “younger” cognitive abilities (Ming and Song, 2005; Glisky, 2007; Trelle et al., 2020; Katsumi et al., 2021).

Next, in this study, we compared how each animal, either young or old, behaves in activities that involve short-term and long-term memories; the result was unexpected. The significant negative correlation between the short- and long-term memories of each animal (the first axis of both PCAs) suggests an age-dependent difference in both memories, with the aged degus having higher values for long-term memory but poor short-term recognition memory. We found the opposite pattern in the young animals. This result suggests that memory becomes segregated by age: young animals with high short-term memory performance tend to show poor long-term memory. Instead, old animals that show poor short-term memory tend to display high long-term memory performance. These data could indicate that older memories are more resistant than novel memories (Cowan, 2008; McGaugh, 2013). Due to the capacity to hold on to information over short periods having a critical role in almost every cognitive task (Liang et al., 2016), reduced short-term memory capacity in aged animals highlights storage difficulty. In accordance with this, studies indicate that older adults exhibit significant deficits in tasks that involve active manipulation, reorganization, or integration of the contents of short-term memory, and that these deficits could be due to impairments in the ability to refresh recently processed information (Glisky, 2007).

Age-dependent differences may have a substrate within the medial temporal lobe, in structures that have different maturation periods; thus, the learning and memory functions emerged differently in time across postnatal development in rodents, nonhuman primates, and humans (Alvarado and Bachevalier, 2000; Reger et al., 2009; McQuail et al., 2012). The lesser long-term memory observed in young degus could have a parallel explanation in the general observation of “infantile amnesia” or the inability of adults to recall infantile memories due to underdeveloped brain (Campbell and Campbell, 1962; Hayne, 2004; Reger et al., 2009; Li et al., 2014). The faster rate of forgetting at a younger age is a well-documented phenomenon across many animal groups. For example, Campbell and Campbell (1962) trained infant and adult rats in an aversively motivated avoidance task (Campbell and Campbell, 1962). When the animals were tested immediately after training, rats of all ages showed high and comparable levels of avoidance. However, as the retention interval increased, they observed marked age differences in performance, with the infant rats exhibiting complete forgetting after 21 days; whereas the adult rats exhibited perfect retention after a longer interval of 42 days. In a similar experiment, Rudy and Morledge (1994) trained rats for contextual fear conditioning, showing that young rats forget more quickly than older rats, thus reflecting a difference in their short-term and long-term memory abilities. A possible explanation could be that young rats have not yet fully developed the neural substrates for a stable long-term memory (Rudy and Morledge, 1994). Reger et al. (2009) showed that weanling rats exhibited robust object recognition memory across shorter delays but lesser long-term memory retention (Reger et al., 2009). Weanling rats performed the novel object recognition task, and exhibited recognition ability nonetheless showed a retention deficit. Similar results from a non-fear-based task were reported for humans (Rovee-Collier and Cuevas, 2009). Human babies trained in a reinforcement task (where the infant learns to kick one leg to move to an overhanging mobile), or in a training task (where the infant learns to press a lever to cause an electric train to move), were slower to learn and faster to forget than adults. These data suggest that these behavioral changes reflected onto genetic changes in memory processes.

It is known that there is a time-dependent consolidation process that will be necessary to stabilize the memory, requiring the participation of full hippocampal formation (Lynch, 2004). The progressive maturation of hippocampal-dependent memory functions during development may reflect the maturity of the functional architecture of the hippocampus, dentate gyrus, and cortical areas that mediate memory. All these structures can contribute differentially to memory formation both in infancy and adulthood. The study of Reuter-Lorenz and Sylvester (2005) reported that different brain areas were activated during working memory tasks in young and old adults, particularly within the prefrontal cortex, suggesting that younger and older adults were performing these tasks differently (Reuter-Lorenz and Sylvester, 2005). This evidence could explain the differences observed between young and aged degus in short- and long-term memories. Although the hippocampus is a common brain area for the consolidation process of both types of memory, the functional network for each includes different adjacent areas whose process of both maturation and aging varies in a multifactorial manner and at different temporal levels (de Oliveira et al., 2011; Jacobs et al., 2015). From an evolutionary point of view, young and adult animals face ecological pressures with adaptive solutions that can be different but equally effective (Martins, 2011; Croft et al., 2015; Capucchio et al., 2019). With aging, ecological demands change, so do their adaptive strategies and the physiological and neural mechanisms that evolve to support them (Austad, 1997; Rovee-Collier and Cuevas, 2009; Martins, 2011). As a result, young and adult animals select to learn different things about the same event, and the younger ones can learn some associations that adults cannot learn at all (Rovee-Collier and Cuevas, 2009).

The differences in short- and long-term memory across ages can also suggest dissociative cellular and molecular components that are more evident under pathological conditions. A mice model of Down syndrome (the Tc1 mice) shows impaired short-term memory but intact long-term memory in the novel object recognition task (Morice et al., 2008). Meanwhile, an AD model with overexpression of APP protein displays regular short-term memory but impaired long-term memory (Good and Hale, 2007; Puri et al., 2015). These data show that the loss of short- or long-term memory in an aged individual could be a sign of a pathological condition.

Aged degus present a pathology with remarkable similarities to that of human late-onset AD (Inestrosa et al., 2005; Cisternas et al., 2018). From a genetic point of view, there are high similarities between human and degus proteins, such as ApoE4, amyloid-β peptide, and *tau* protein (Salazar et al., 2016; Steffen et al., 2016; Hurley et al., 2018). However, two studies questioned the validity of degus as a valuable model for AD research (Bourdenx et al., 2016; Steffen et al., 2016). A possible explanation for their findings is that the small group of animals per group they used was not assessed for AD-like behavioral changes before the neuropathology analysis was conducted (Hurley et al., 2018). Moreover, it also cannot be ruled out that the presence of the ApoE gene in degus may vary between colonies. Many colonies of degus are derived from laboratory-bred animals compared to laboratory-born animals mated with wild-trapped degus. It is precisely in the latter that behavioral changes correlate with AD-like pathology. Regarding this issue, Hurley et al. (2018) suggested that degus bred in the laboratory do not suffer comorbid diseases, natural stresses, and consequent epigenetic effects. More experiments are needed to prove this statement.

In general, the results are in agreement with those of previous studies on degus, where an age-dependent decline in memory performance begins at 36-months of age and persists throughout old age (Ardiles et al., 2012). However, the results differ from those in Ardiles et al., which reported that about 25% of 36-months-old degus exhibit unimpaired performance on the behavioral tasks (Ardiles et al., 2012). In this study, only 5% of young and 80% of aged degus showed impairment in their short-term memory (IR lower than 0.55), and for long-term memory, about 32% of young animals and 57% of aged degus showed impairment in their performance on the Barnes maze test. Like humans, the natural variability in cognitive performance observed across the ages of the animals included in this study should be expected in this long-lived animal model. Taken together, this information could provide important clues to understand how aging affects learning and memory differently and help us detect early age-related memory impairments based on behavioral performance.

Throughout this study, we showed that long-lived degus are a fruitful model for understanding the dissociations in behavioral performance associated with the natural aging process. Moreover, we also demonstrated that using the multivariate approach let us explore, correlate, and visualize several complex behavioral variables related to cognition, learning, and memory in a macroscale fashion. More importantly, we can also identify specific behavioral patterns that allow us to make robust conclusions than if the variables were analyzed independently. On the other hand, social behavior (i.e., interaction and approach), short-term recognition memory, and long-term spatial memory (formation and consolidation) are all under the regulation of several brain regions (Hitti and Siegelbaum, 2014; Garrido Zinn et al., 2016; Tanimizu et al., 2017). Further studies should include additional variables such as neural and molecular markers or physiological measurements to fully understand the impact of natural aging or pathological aging (such as AD) involved in both short- and long-term memories in this animal model.

## Data Availability Statement

Publicly available datasets were analyzed in this study. The information on cognitive performance included published and unpublished data obtained in the last six years in our laboratory. Further inquiries can be directed to the corresponding authors.

## Ethics Statement

The animal study was reviewed and approved by Bioethical and Biosafety Committee of the Faculty of Biological Sciences of the Pontificia Universidad Católica de Chile (CBB-121-2013).

## Author Contributions

DR: conceptualization, formal analysis, data curation, writing—original draft, review and editing, visualization, and funding acquisition. CL and CO: writing—review and editing and visualization. FB and NI: writing—review and editing, supervision, and funding acquisition. All authors contributed to the article and approved the submitted version.

## Conflict of Interest

The authors declare that the research was conducted in the absence of any commercial or financial relationships that could be construed as a potential conflict of interest.

## Publisher's Note

All claims expressed in this article are solely those of the authors and do not necessarily represent those of their affiliated organizations, or those of the publisher, the editors and the reviewers. Any product that may be evaluated in this article, or claim that may be made by its manufacturer, is not guaranteed or endorsed by the publisher.
