# Supplementary material for: A Multivariate Assessment of Age-Related Cognitive Impairment in Octodon degus
Source: Front Integr Neurosci. 2021 Aug 30;15:719076. doi: 10.3389/fnint.2021.719076 (PMC8437396; doi:10.3389/fnint.2021.719076)
Supplement: Supplementary file 1 [file Data_Sheet_1.docx]

**SUPPLEMENTARY MATERIAL**

**Supplementary Table 1.** Rotated component patterns for the Open Field test. Only component patterns above 0.40 were recorded.

| Variables | C1 | C2 |
| --- | --- | --- |
| Time spent in the central zone  Time spent in the corners  Number of central crossings  Total distance traveled  Speed | -0.73  0.77  0.52  0.87  0.87 | 0.55  -0.50  **-**  **-**  **-** |

**Supplementary Table 2**. Rotated component patterns for the short-term memory measured by the Social Interaction and the Novel Local/Object Recognition (NLR/NOR) tests. Only component patterns above 0.40 were recorded.

| Variables | C1 |
| --- | --- |
| RI of the Social Interaction test  RI of NLR test  RI of NOR test | 0.79  0.75  0.84 |

**Supplementary Table 3**. Rotated component patterns for the long-term memory measured during the test phase of the Barnes maze test. Only component patterns above 0.40 were recorded.

| Variables | C1 | C2 |
| --- | --- | --- |
| Latency to the first visit of the escape hole  Reference memory errors to find the escape hole  Working memory errors to find the escape hole | -  0.84  0.92 | 0.97  -0.46  **-** |

**Figure legends**

**Supplementary Figure 1.** Principal component analysis (PCA) graph of the Open Field test across age groups. Each symbol represents the age of animals (circle: 12-months old (n = 6 per group); square: 24-months old (n = 13 per group); triangle: 38-months old (n = 6 per group); diamond: 56-months old (n = 5 per group); inverted triangle: 75-months old (n = 9 per group). We used the time that animals spent in the central zone, the time spent in the corners, the number of central crossings, the speed, and the total distance traveled during the Open Field test. The F and p values of the one-way PERMANOVA test are plotted.

**Supplementary**
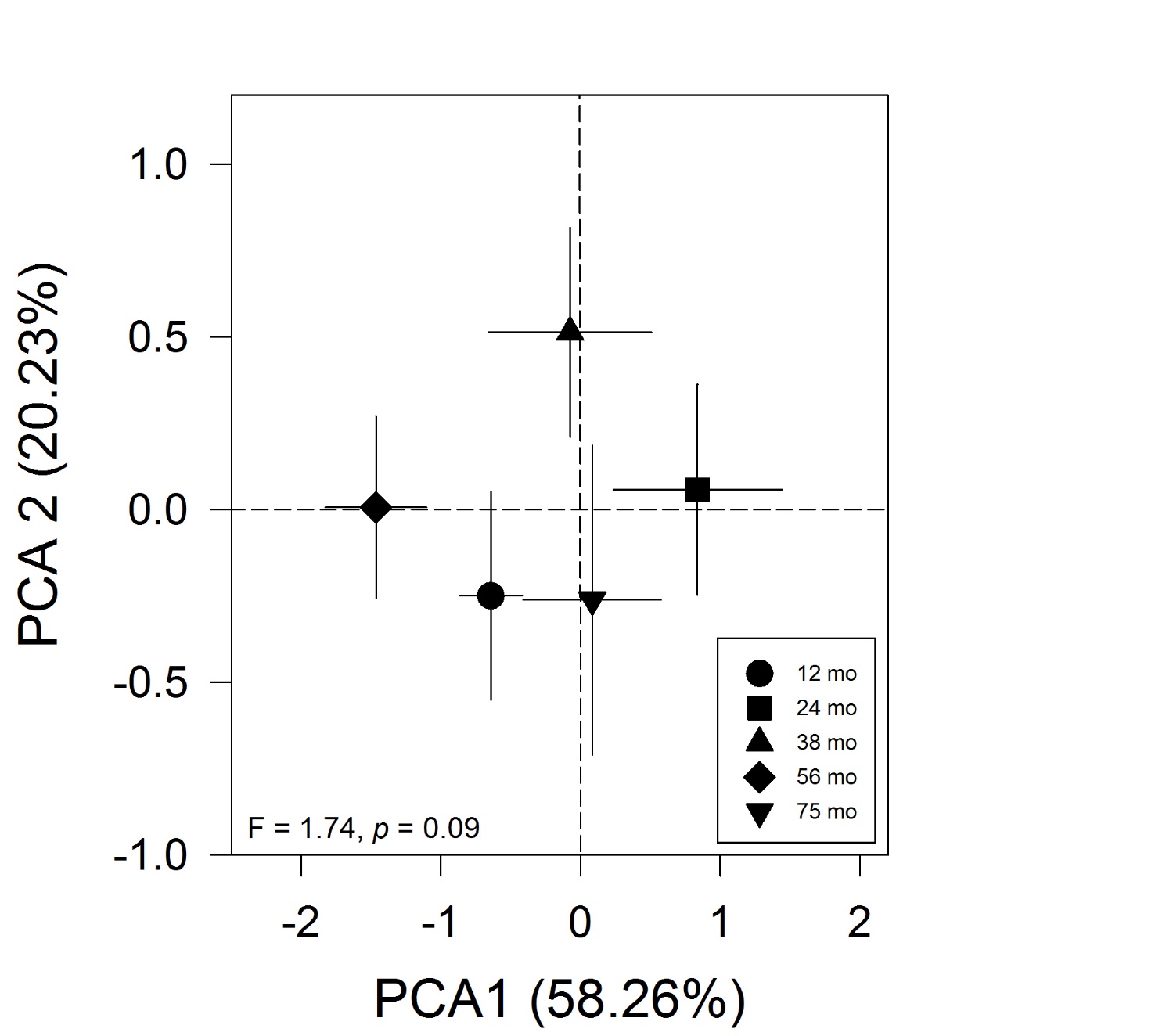
**Figure 1**
